# Mapping the current state of pediatric surgical pain care across Canada and assessing readiness for change

**DOI:** 10.1080/24740527.2022.2038031

**Published:** 2022-06-06

**Authors:** Kathryn A. Birnie, Jennifer Stinson, Lisa Isaac, Jennifer Tyrrell, Fiona Campbell, Isabel P. Jordan, Justina Marianayagam, Dawn Richards, Brittany N. Rosenbloom, Fiona Clement, Pam Hubley

**Affiliations:** a Department of Anesthesiology, Perioperative, and Pain Medicine, University of Calgary, Calgary, Alberta, Canada; b Department of Community Health Sciences, University of Calgary, Calgary, Alberta, Canada; c Alberta Children’s Hospital Research Institute, Calgary, Alberta, Canada; d Child Health Evaluative Sciences, The Hospital for Sick Children, Toronto, Ontario, Canada; e Lawrence S. Bloomberg Faculty of Nursing, University of Toronto, Toronto, Ontario, Canada; f Department of Anesthesia and Pain Medicine, The Hospital for Sick Children, Toronto, Ontario, Canada; g Department of Anesthesiology and Pain Medicine, University of Toronto, Toronto, Ontario, Canada; h Parent Partner, Partnering For Pain Research Program, University of Calgary; i Northern Ontario School of Medicine, Thunder Bay, Ontario, Canada; j Five02Labs, Inc, Toronto, Ontario, Canada; k Education, Academic Practice & Patient Experience, The Hospital for Sick Children, Toronto, Ontario, Canada

**Keywords:** children, surgery, pediatric pain, postsurgical pain, health systems, care delivery

## Abstract

**Background:**

Preventing pediatric chronic postsurgical pain is a patient, parent/caregiver, health care professional, and policymaker priority. Poorly managed presurgical and acute postsurgical pain are established risk factors for pediatric chronic postsurgical pain. Effective perioperative pain management is essential to prevent the transition from acute to chronic pain after surgery.

**Aims:**

The aim of this study was to identify current pediatric surgical pain management practices and assess health system readiness for change at health care institutions conducting pediatric surgery in Canada.

**Methods:**

An online survey was completed by 85 multidisciplinary health care professionals (nurses, surgeons, anesthesiologists, allied health) from 20 health institutions in Canada regarding institutional pre- and postsurgical pediatric pain care, specialty pain services, and Organizational Readiness for Implementing Change (ORIC).

**Results:**

Of all specialty pain services, acute and chronic/complex pain services were most common, primarily with physician and nursing involvement. Alignment to recommended practices for pediatric pre- and postsurgical pain care varied (38.1%–79.8% reported “yes, for every child”), with tertiary/quaternary children’s hospitals reporting less alignment than other institutions (community/regional or rehabilitation hospitals, community treatment centers). No significant differences were reported between health care institutions serving pediatric populations only versus those also serving adults. Health care professional experience/practice was the most reported strength in pediatric surgical pain care, with inconsistent standard of care the most common gap. Participants “somewhat agreed” that their institutions were committed and capable of change in pediatric surgical pain care.

**Conclusions:**

There is a continued need to improve pediatric pain care during the perioperative period at Canadian health care institutions to effectively prevent the development of pediatric postsurgical pain.

## RÉSUMÉ

**Contexte**: La prévention de la douleur post-chirurgicale chronique pédiatrique est une priorité des patients, des parents/prestataires de soins, des professionnels de la santé et des décideurs. La douleur pré-chirurgicale et post-chirurgicale aiguë mal prises en charge constituent des facteurs de risque connus de la douleur post-chirurgicale chronique pédiatrique. La prise en charge efficace de la douleur périopératoire est essentielle pour prévenir la transition de la douleur aiguë à la douleur chronique aprés une chirurgie.

**Buts**: Le but de cette étude était de recenser les pratiques actuelles en matiére de prise en charge de la douleur chirurgicale pédiatrique et d’évaluer l'état de préparation au changement du systéme de santé dans les établissements de soins de santé menant des chirurgies pédiatriques au Canada.

**Méthodes**: 85 professionnels de la santé multidisciplinaires (infirmiéres, chirurgiens, anesthésistes, auxiliaires médicaux) ont répondu à un sondage en ligne de 20 établissements de santé au Canada concernant les soins pédiatriques pré et post-chirurgicaux en établissement, les services spécialisés de traitement de la douleur, les services de la douleur spécialisés et la préparation organisationnelle à la mise en œuvre du changement.

**Résultats**: De tous les services de la douleur spécialisés, les services de la douleur aiguë et chronique/complexe étaient les plus fréquents, principalement avec la participation d'un médecin et d'une infirmiére. L’adéquation avec les pratiques recommandées pour les soins pédiatriques pré et post-chirurgicaux était variable (38,1 %-79,8 % ont déclaré « oui, pour chaque enfant »), alors que les hôpitaux pour enfants tertiaires/quaternaires ont déclaré moins d'adéquation que les autres institutions (hôpitaux communautaires/régionaux ou de réadaptation, centres de traitement communautaires). Aucune différence significative n’a été rapportée entre les établissements de soins de santé desservant uniquement les populations pédiatriques et ceux desservant aussi les adultes. L'expérience professionnelle/la pratique en matière de soins de santé a été la force la plus rapportée dans les soins de la douleur chirurgicale pédiatrique, tandis que les normes de soins incoh'rentes constituaient la lacune la plus courante. Les participants ont « quelque peu convenu » que leurs institutions étaient engagées et capables de changer les soins chirurgicaux pédiatriques.

**Conclusions**: ll y a un besoin continu déaméliorer les soins de la douleur pédiatrique pendant la période périopératoire dans les établissements de soins de santé canadiens afin de prévenir efficacement le développement de la douleur postopératoire.

More than 80,000 children in Canada undergo surgery each year.^1^ In 2018–2019, greater than 25,000 children 0 to 17 years old in Canada underwent one of the top ten most common pediatric inpatient surgeries, with average inpatient stays of 1.4 to 68.2 days.^2^ Of those children and adolescents undergoing major surgery (i.e., surgeries requiring an inpatient stay), an estimated 20% will develop chronic postsurgical pain (i.e., pain lasting 3 months or more).^3,4^ Risk factors for pediatric chronic postsurgical pain after major surgeries include presurgical child anxiety, pain coping efficacy, pain intensity and disability, parental pain catastrophizing, and postsurgical opioid consumption and acute pain.^3,5–9^ Moreover, 2.7% to 15.2% of previously opioid-naïve youth have continued to use opioids prescribed after varied surgeries long term (i.e., >90 days after surgery).^10^ Opioids prescribed for pain management in adolescence place youth at increased risk for subsequent opioid use problems that can persist in adulthood.^11–13^

The top patient-oriented research priority for pediatric chronic pain is to prevent acute pain from becoming chronic.^14,15^ Given that surgery is a known potential risk factor for new-onset chronic pain, effective pain management during the perioperative period provides a unique opportunity to prevent the development of chronic pain. Data from the United States indicate that the top 20 pediatric inpatient surgical procedures account for 80% of all surgeries for children <18 years of age, of which 40% are performed in adult general hospitals.^16^ As such, it is important that perioperative pain care be considered in both specialized children’s hospital, as well as in community or regional hospitals where both pediatric and adult populations are treated. Preventing chronic postsurgical pain is also a top strategic priority for provincial, territorial, and federal governments to improve chronic pain care^17,18^ and for adult surgical patients, their caregivers, and health care professionals.^19^

Several best practices and clinical practice guidelines have emerged for pediatric surgical pain care, with some addressing a multimodal approach to pain management (pharmacological, psychological, physical strategies)^20^ and others focused on opioids.^21,22^ These guidelines include recommendations for health care professional practices with implications for health system design, for children and their parents/caregivers, and across hospital and community or home settings. Recommendations for pediatric surgical pain care include activities to be undertaken prior to surgery (i.e., education for children and parents/caregivers about pain, co-development of a postoperative pain management plan, and assessment of psychosocial risk factors for postoperative pain), as well as actions after surgery (i.e., pain assessment, multimodal pain management, safe and effective use of opioids, and discharge planning for pain management at home).^20,23^ Despite the preliminary application of a measure to identify children at risk for chronic postsurgical pain^24^ and the availability of treatment guidelines,^20^ there is no established model of care targeting the prevention of pediatric chronic postsurgical pain.^20,25^ This is critical to address because under treatment of pediatric postoperative pain is widespread^25,26^ and can delay remobilization and negatively impact health-related quality of life, including problems with sleep, anxiety, social, family, and school functioning.^23,27–30^

Transitional pain services have emerged in adult tertiary care as an innovative and effective health service model to prevent the transition from acute to chronic pain after surgery.^31^ In transitional pain services, patients at high risk for chronic postsurgical pain are identified early (preoperatively or in-hospital postoperatively) and are offered coordinated and comprehensive multidisciplinary outpatient care from pain physicians, nurses, psychologists, and physiotherapists.^32^ Transitional pain services have demonstrated improvements for chronic postsurgical pain risk factors of pain, disability, pain catastrophizing, anxiety, depression, and opioid use in adults.^31,33,34^ Despite the existence of transitional pain services for pediatric populations,^35^ to our knowledge no published evaluation of their implementation or effectiveness exists. Furthermore, though existing adult transitional pain services models offer some guidance for pediatric care, the need for developmentally tailored pain services for pediatric patients, including the involvement of parents/caregivers, is well recognized.^36^

Successful implementation of scientific evidence in health system design depends on the degree to which it is embedded in the daily experience and practice of its users (patients, families, and health care professionals).^37^ The primary goal of this project was to survey children’s health care centers across Canada to identify current practices and alignment with pediatric surgical pain care recommendations,^20,23^ as well as health system readiness for change. This patient-oriented study forms the first of three phases in a human-centered design^38^ project seeking to co-design pediatric pain services to prevent the development of chronic postsurgical pain in children and adolescents. Exploratory aims added post hoc were to explore differences in pediatric surgical pain care by type of health care institution (i.e., institutions serving just pediatric populations versus life span and tertiary/quaternary children’s hospitals versus other institutions). Given that these exploratory aims were added post hoc based on guidance from the project team, including people with lived experience, no hypotheses were specified.

## Materials and Methods

### Study Design

An online survey assessed current service delivery models and practices for managing pediatric pain for surgery, as well as health system readiness for change in surgical pediatric pain care at diverse health care institutions across Canada. This patient-oriented research study engaged people with lived experience as equal members of the research team, including in designing study aims, methods (survey development and refinement), analysis, interpretation of study findings, and co-authorship of this article.

### Participants

Eligible participants included any health care professionals and administrators working in and/or overseeing acute, chronic, perioperative pain or surgical services involving children <18 years old within a children’s health care institution in Canada. A children’s health care institution was defined as an organization that delivers health services to children and adolescents (and may also deliver health services to adults). Individuals were excluded if they worked outside of Canada and/or worked exclusively with adults (i.e., individuals >18 years old). Multiple respondents were eligible from the same health institution.

### Study Procedure

This project was approved by the Conjoint Health Research Ethics Board at the University of Calgary (REB20-1578). The online survey was open from June 2 to 26, 2021. The online survey was distributed through (1) direct e-mail invitation to eligible health care professionals and administrators known to the research team members and/or identified through relevant program websites online (i.e., via web search for surgical program leads at institutions across Canada), (2) Children’s Healthcare Canada membership e-mail distribution lists and newsletter, (3) posting to relevant professional listservs (Pain in Child Health), (4) social media postings by the team members and project partners (Solutions for Kids in Pain), and (5) posting recruitment information to our lab website (www.partneringforpain.com). A snowball recruitment method was used, whereby individuals were encouraged to pass on the e-mail and study invitation to relevant colleagues in their networks. The online survey was administered through REDCap.^39^

Informed consent was completed electronically as part of the online survey prior to the eligibility screening questions and subsequent survey completion. Consent was implied through selecting “yes” to consenting to the study as part of the first page of the survey and then indicated by subsequent completion of the survey questions. Study eligibility screening was done through screening questions following informed consent. If survey respondents were not eligible, the survey ended; otherwise, eligible participants were able to complete subsequent survey questions. At the end of the survey, participants had the option of entering their e-mail address to be entered into a drawing to win one of four $50 gift cards.

### Online Survey Questions and Analysis

The online survey contained a total of 41 multiple-choice, numerical rating, or open-ended questions co-developed and refined by the research team, including patient partners (see Supplementary Table 1 for survey questions). The survey included demographic questions (e.g., professional role[s], year of birth, sex, gender, years working in child health, etc.), information about the health care institution (e.g., size, type of surgical care provided, current pediatric pain services), 12 questions regarding alignment of pain services to existing best practice recommendations for preventing and managing acute postsurgical pain with mention of relevance to pediatric populations (e.g., use of psychological, pharmacological, and physical strategies; provision of pain education; etc.), and pediatric pain literature regarding risk factors for chronic postsurgical pain,^3,20^ as well as open-ended questions regarding perceived strengths and gaps in pain management for children undergoing surgery at their health care institution.

The final 12 questions of the survey were adapted from the Organizational Readiness for Implementing Change (ORIC) questionnaire.^40^ Organizational readiness is an important factor in successful implementation of new health care policies, programs, and practices. All items in the ORIC are responded to on a 5-point Likert scale: *agree, somewhat agree, neither agree nor disagree, somewhat disagree, disagree*. Exploratory and confirmatory factor analyses revealed that the ORIC assesses two subscales of organizational readiness: change commitment and change efficacy.^40^

The online survey data were summarized using descriptive statistics (e.g., frequencies, means/standard deviations) as appropriate. Open-ended questions were coded using simple content analysis.^41,42^ All responses were read by two coders (lead author K.A.B. and a research assistant) to become familiar with responses provided. Codes were developed to stay close to the surface of the words used in the open-ended responses with minimized interpretation. One author (K.A.B.) coded the open-ended responses.

## Results

### Participants and Health Care Institutions

A total of 164 respondents began the survey. Of these, 5 were not eligible as per the screening questions, 41 consented but did not complete the survey, and 118 consented. Of these, 33 (28.0%) respondents worked at health care institutions that serve children and adolescents but did not conduct surgery and so were not included. The remaining 85 (72.0%) worked at a health care institution that conducted pediatric surgery. Reported results are based on this sample of 85 participants.

See Table 1 for participant demographic information. See Table 2 for the list of participants’ health care institutions across Canada and Table 3 for detailed characteristics about the health institutions.

**Table 1.** Participant demographic characteristics.

Demographic characteristics	Mean (SD)	*n* (%)
Professional role(s)		
Nurse		17 (20.0)
Surgeon		16 (18.8)
Nurse practitioner		15 (17.6)
Clinical nurse specialist		12 (14.1)
Nurse educator		10 (11.8)
Manager or administrator		9 (10.6)
Anesthesiologist		9 (10.6)
Allied health (e.g., occupational therapist, child life therapist, physical therapist, psychologist) Other physician (e.g., pediatrician)		8 (9.4) 2 (2.4)
Clinical programs you are part of and/or oversee		
Surgical services or programs—Ambulatory		26 (30.6)
Surgical services or programs—Major surgeries requiring inpatient stay		40 (47.1)
Medical services or programs		19 (22.4)
Acute pain service		20 (23.5)
Transitional pain service		9 (10.6)
Chronic/complex pain service		14 (16.5)
Intensive pain rehabilitation service		3 (3.5)
Time in current role (years)	7.79 (6.51)	
Age (years)	38.40 (11.51)	
Gender		
Female		63 (74.1)
Male		22 (25.9)
Race		
White		79 (92.9)
Indigenous (e.g., Aboriginal, First Nations, Inuit, Metis)		3 (3.5)
Latin American		1 (1.2)
Chinese		1 (1.2)
Arab		1 (1.2)
West Asian (e.g., Iranian, Afghan)		1 (1.2)
Black		1 (1.2)
Declined to state/missing		2 (2.4)
Perceived or treated as a person of color		
Yes		3 (3.5)
No		82 (96.5)

**Table 2.** Health care institutions.

Participants’ health care institutions in Canada
Alberta Children’s Hospital
BC Children’s Hospital
Center Hospitalier de l’Universite de Sherbrooke
CHEO (Children’s Hospital of Eastern Ontario)
Children’s Hospital–Health Sciences Center (Winnipeg, MB) Children’s Hospital at London Health Sciences Center Children’s Treatment Network of Simcoe York
Chinook Regional Hospital
CHU Sainte-Justine
Holland Bloorview Kids Rehabilitation Hospital
IWK Health Center
Jim Pattison Children’s Hospital
McMaster Children’s Hospital
Regina General Hospital
Shriners Hospital for Children Canada
Stollery Children’s Hospital
The Hospital for Sick Children
The Janeway Children’s Hospital & Rehabilitation Center
Thunder Bay Regional Health Sciences Center
Victoria General Hospital

**Table 3.** Health care institution characteristics.

Institution characteristics	*n* (%)
Type of patient population(s)	
Pediatric only	56 (65.9)
Pediatric and adult	29 (34.1)
Type of children’s health institution	
Tertiary/quaternary children’s hospital	46 (54.1)
Rehabilitation hospital	9 (10.6)
Community/regional hospital	18 (21.2)
Children’s community treatment center	12 (14.1)
Number of pediatric inpatient beds	
1–50	31 (36.5)
51–100	15 (17.6)
101–150	8 (9.4)
151–200	10 (11.8)
200+	17 (20.0)
Unsure	4 (4.7)
Type of specialty pain service(s)	
None	4 (4.7)
Acute pain service	50 (58.8)
Transitional pain service	30 (35.3)
Chronic/complex chronic pain service	51 (60.0)
Intensive pain rehabilitation service	13 (15.3)
Type of pediatric surgeries	
Ambulatory (day surgery) Number per year	61 (71.8)
1–50	13 (21.3)
51–100	5 (8.2)
101–150	1 (1.6)
151–200	0 (0)
200+	24 (39.3)
Unsure	18 (29.5)
Major (requiring inpatient stay)	66 (77.6)
Number per year	
1–50	11 (17.2)
51–100	4 (6.3)
101–150	2 (3.1)
151–200	3 (4.7)
200+	27 (42.2)
Unsure	17 (26.6)

### Reported Pain Services at Health Care Institutions

Most participants agreed (*n* = 53; 62.5%) or somewhat agreed (*n* = 28; 32.9%) that pain was a priority for their health care institution. Fewer participants neither agreed nor disagreed (*n* = 2; 2.4%) or somewhat disagreed (*n* = 2; 2.4%) that pain was a priority for their health care institution. No respondents disagreed.

See Table 3 for type of specialty pediatric pain services at participants’ health care institutions. More than half of participants reported working at a health care institution with a chronic/complex pain service (i.e., outpatient/ambulatory multidisciplinary clinic) and/or an acute pain service (i.e., inpatient consultations), with fewer reporting having a transitional pain service (i.e., predominantly outpatient that serves children at risk for developing chronic pain) or an intensive pain rehabilitation program (i.e., outpatient and/or inpatient). Only a small portion of participants reported that their health care institution had no specialty pain service.

#### Acute Pain Services

For those participants at institutions with an acute pain service (*n* = 50; 58.8%), the majority reported having physician (*n* = 44; 88.0%) and nursing involvement (*n* = 40; 81.0%), with fewer reporting physical therapy (*n* = 8; 16.0%), child life (*n* = 8; 16.0%), occupational therapy (*n* = 6; 12.0%), psychology (*n* = 6; 12.0%), recreational therapy (*n* = 1; 2.0%), family therapy (*n* = 1; 2.0%), or pharmacy (*n* = 1; 2.0%) as members of the acute pain team. A portion (*n* = 6; 12.0%) were unsure as to health care professionals on the acute pain service team.

#### Transitional Pain Services

For those participants at institutions with a transitional pain service (*n* = 30; 35.3%), less than half reported having a physician (*n* = 14; 46.7%) and nursing involvement (*n* = 13; 43.3%), followed by physical therapy (*n* = 11; 36.7%), psychology (*n* = 10; 33.3%), occupational therapy (*n* = 9; 30.0%), family therapy (*n* = 2; 6.7%), recreational therapy (*n* = 2; 6.7%), and child life (*n* = 1; 3.3%) as members of the transitional pain service. A portion (*n* = 5; 16.7%) were unsure as to health care professionals on the transitional pain service team.

#### Chronic/Complex Pain Services

For those participants at institutions with a chronic or complex pain service (*n* = 51; 60.0%), the majority reporting having nursing involvement (*n* = 32; 62.7%), followed by a physician (*n* = 28; 54.9%), physical therapy (*n* = 24; 47.1%), psychology (*n* = 23; 45.1%), occupational therapy (*n* = 15; 29.4%), family therapy (*n* = 4; 7.8%), child life (*n* = 4; 7.8%), recreational therapy (*n* = 3; 5.9%), 9.1%), or pharmacy (*n* = 1; 2.0%) as members of the chronic/complex pain team. A portion (19.6%) were unsure as to health care professionals on the chronic/complex pain service team.

#### Intensive Pain Rehabilitation Services

For those participants at institutions with an intensive pain rehabilitation service (*n* = 13; 15.3%), the majority reporting having physician (*n* = 9; 69.2%), nursing (*n* = 9; 69.2%), psychology (*n* = 9; 69.2%), occupational therapy (*n* = 7; 53.8%), physical therapy (*n* = 7; 53.8%), child life (*n* = 5; 38.5%), (43.8%), family therapy (*n* = 5; 38.5%), recreational therapy (*n* = 4; 30.8%), or art therapy (*n* = 4; 30.8%) as members of the team. A portion (*n* = 3; 23.1%) were unsure as to health care professionals on the intensive pain rehabilitation team.

### Perceived Standard of Pain Care for Pediatric Patients Undergoing Surgery

**Figure 1.**
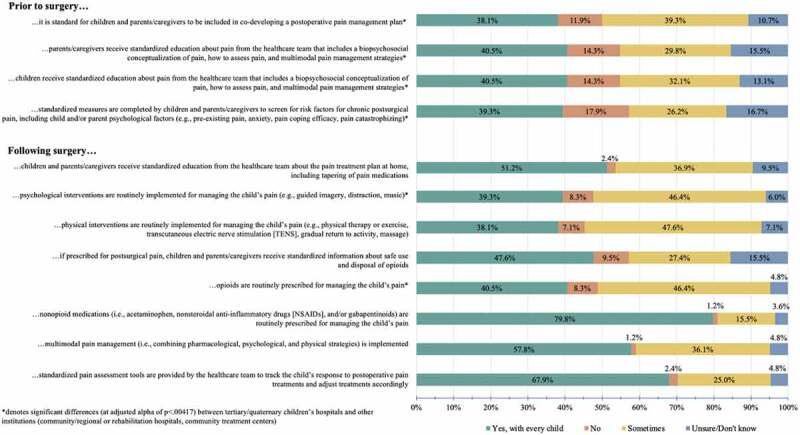
Percentage of participants reporting alignment of health care institution pre- and postsurgical pain practices to recommended practices for preventing and managing chronic postsurgical pain.

See Table 3 for type and number of pediatric surgeries conducted at participants’ health care institutions. See Figure 1 for percentages of participants reporting alignment of health care institution pre- and postsurgical pain practices to existing literature regarding risk factors for pediatric chronic postsurgical pain and recommendations for preventing and managing chronic postsurgical pain. Overall, postsurgical pain care recommendations were more consistently implemented for every child than presurgical recommendations; however, large variability was noted. Across recommendations, 38.1% to 79.8% of participants indicated that recommended surgical pain care practices are implemented for every child, 15.5% to 47.6% indicated they are sometimes implemented, 1.2% to 17.9% indicated they are not implemented, and 3.6% to 16.7% indicated they were unsure or did not know. Less than 5% of participants provided additional comments to response categories and noted that pediatric surgical pain practices were dependent on the individual child and/or the health care professional.

#### Perceived Institutional Strengths in Pediatric Surgical Pain Management

One quarter (25.9%) of participants provided no additional open-ended comment. Coded open-ended responses revealed perceived strengths to be the experience or practices of health care professionals (31.8%), having a relevant specialized pediatric pain team (14.1%; i.e., acute and/or transitional pain services), strong institutional commitment to pediatric pain management (9.4%; e.g., through quality improvement initiatives, leadership support, pain as a priority), implementation of preoperative practices (8.2%; e.g., pain education for families, assessment of risk factors), use of multimodal pain strategies and/or a multidisciplinary approach (7.1%), effective use of pharmacological strategies, (7.1%), pain assessment (5.9%), strong coordinated care transitions (3.6%; i.e., between inpatient units, transition to home), and strong partnership with the patient and/or family (2.4%). A portion of participants (10.6%) made other comments (e.g., being unsure) or with insufficient detail to code further (e.g., simply stating “yes”).

#### Perceived Institutional Gaps in Pediatric Surgical Pain Management

Most participants (84.7%) provided additional open-ended comments. Coded open-ended responses revealed that 23.5% of participants reported no perceived gaps; however, perceived gaps included inconsistent standard of care (14.1%), lack of multimodal or multidisciplinary pain management (12.9%; e.g., no access to psychology or physical therapy), inexperience of health care professionals (9.4%), challenges with medication weaning (9.4%), poorly coordinated care transitions (5.9%; i.e., between inpatient units, transition to home), lack of engagement with patients and families (5.9%; e.g., no pain education provided), lack of resources or staff (5.9%), lack of preoperative pain management planning (e.g., 4.7%), inconsistent pain assessment (3.5%), poor opioid stewardship (3.5%), no specialized pain services (3.5%; e.g., acute and/or transitional pain services), lack of and/or poor technology (3.5%), lack of institutional commitment and/or leadership (2.4%), and inconsistent charting in electronic health records (2.4%). A portion of participants (3.5%) made other comments (e.g., being unsure) or with insufficient detail to code further (e.g., simply stating “yes”).

### Organizational Readiness for Implementing Change in Pediatric Surgical Pain Care

Most participants either agreed (*n* = 41; 48.2%) or somewhat agreed (*n* = 37; 43.5%) that pain was a priority for surgical services at their health care institution. A portion of respondents neither agreed nor disagreed (*n* = 5; 5.9%), somewhat disagreed (*n* = 1; 1.2%), or disagreed (*n* = 1; 1.2%) that pain was a priority for surgical services at their health care institution.

With regards to organizational readiness to implement change to improve the prevention and management of pain for children and adolescents undergoing surgery, participants’ overall mean ratings were close to “somewhat agree” for both change commitment (*M* = 1.80; SD = 0.65; 1 = *agree* to 4 = *somewhat disagree*) and change efficacy (*M* = 1.99; SD = 0.71; 1 = *agree* to 4 = *somewhat disagree*).

### Differences by Type of Health Care Institution

#### Pediatric versus Life Span Populations Served

Pediatric-only health care institutions may have different resources, policies, and/or practices for surgical and/or pediatric pain care than those serving both pediatric and adult populations. At the recommendation of research team members, including patient partners, additional exploratory analyses were conducted comparing standard of pain care and organizational readiness for change in pediatric surgical pain care between health care institutions serving pediatric populations only (*n* = 56; 65.9%) and those serving both pediatric and adult populations (*n* = 29; 34.1%). Given that these are exploratory analyses, we applied a more stringent alpha correction threshold for statistical significance of *P* < 0.00417 (0.05/12 post hoc analyses).

Chi-square analyses revealed no significant differences between health care institutions serving pediatric populations only and those additionally serving adult populations on alignment with the 12 pre- and postsurgical pain management practices.

Participants from health care institutions serving only pediatric populations were no more likely to report “no,” “sometimes,” or “don’t know/unsure” compared with “yes, for every child” to implementation of preoperative completion of standardized measures by children and parents/caregivers for risk factors for chronic postsurgical pain, χ^2^(3) = 9.34, *P* = 0.025. No significant group differences were found for children, χ^2^(3) = 5.01, *P* = 0.171, or parents/caregivers, χ^2^(3) = 5.56, *P* = 0.135, receiving standardized education about pain prior to surgery from the health care team or standard preoperative practice for children and parents/caregivers to be included in co-developing a postoperative pain management plan, χ^2^(3) = 4.03, *P* = 0.258.

No significant differences were reported by participants from health care institutions serving only pediatric populations versus institutions serving pediatric and adult populations in any postoperative pain management practices, including routine prescription of nonopioid medications after surgery, χ^2^(3) = 6.61, *P* = 0.086; routine prescription of opioids for managing pain after surgery, χ^2^(3) = 1.73, *P* = 0.630; providing children and parents/caregivers with standardized information about use and disposal of opioids when prescribed, χ^2^(3) = 4.92, *P* = 0.178; routinely implementing physical interventions, χ^2^(3) = 4.45, *P* = 0.217, or psychological interventions, χ^2^(3) = 0.76, *P* = 0.860; providing standardized education about the pain treatment plan at home, χ^2^(3) = 6.83, *P* = 0.078; standardized pain assessment to track response to postoperative pain treatments or adjust treatments accordingly, χ^2^(3) = 2.45, *P* = 0.485; or routine implementation of multimodal pain management, χ^2^(3) = 5.23, *P* = 0.156.

Independent sample *t* tests revealed no significant group differences in change commitment or change efficacy for organizational readiness to improve the prevention and management for children undergoing surgery between health care institutions serving pediatric populations only (change commitment: *M* = 1.73; SD = 0.61; change efficacy: *M* = 1.95; SD = 0.70) and institutions serving both pediatric and adult populations (change commitment: *M* = 1.94; SD = 0.71), *t*(82) = −1.42, *P* = 0.160 (change efficacy: *M* = 2.05; SD = 0.75), *t*(81) = −.59, *P* = 0.559).

#### Tertiary/Quaternary Children’s Hospitals versus Other Institutions

Tertiary/quaternary children’s hospitals are more likely than other health care institutions to be conducting pediatric surgeries and in larger numbers. As such, additional exploratory analyses were conducted to see whether differences in standard of pain care and organizational readiness for change in pediatric surgical pain care differed between tertiary/quaternary children’s hospitals (*n* = 46; 54.1%) and other health care institutions (i.e., rehabilitation hospitals, community/regional hospitals, community treatment centers; *n* = 39; 45.9%). As above, we applied a more stringent alpha correction threshold for statistical significance of *P* < 0.00417 (0.05/12 post hoc analyses).

Chi-square analyses revealed significant differences between tertiary/quaternary children’s hospital and other health care institutions alignment on all 12 pre- and postsurgical pain management practices.

Participants from tertiary/quaternary children’s hospitals were significantly more likely to report “no,” “sometimes,” or “don’t know/unsure” compared with participants from other institutions, who more frequently reported “yes, for every child” to implementation of preoperative pain management practices, including: completion of standardized measures by children and parents/caregivers for risk factors for chronic postsurgical pain, χ^2^(3) = 32.24, *P <* 0.0001, children receiving standardized education about pain from the health care team, χ^2^(3) = 16.71, *P* = 0.001, parents/caregivers receiving standardized education about pain from the health care team, χ^2^(3) = 13.47, *P* = 0.004, or that it is standard for children and parents/caregivers to be included in co-developing a postoperative pain management plan, χ^2^(3) = 26.72, *P* < 0.0001. Following surgery, participants from other institutions versus tertiary/quaternary children’s hospitals were more likely respond “yes, for every child” regarding routinely prescribing opioids, χ^2^(3) = 16.88, *P* = 0.001, and implementing psychological interventions, χ^2^(3) = 28.16, *P* < 0.0001.

No significant differences were reported by participants from tertiary/quaternary children’s hospital versus other institutions in postsurgical pain assessment to track response to postoperative pain treatments or adjust treatments accordingly, χ^2^(3) = 3.80, *P* = 0.284, routine implementation of multimodal pain management, χ^2^(3) = 1.71, *P* = 0.636, implementation of physical interventions, χ^2^(3) = 9.01, *P* = 0.029, routine prescription of nonopioid medications after surgery, χ^2^(3) = 10.55, *P* = 0.014, providing children and parents/caregivers with standardized information about use and disposal of opioids when prescribed, χ^2^(3) = 12.59, *P* = 0.006, or providing standardized education about the pain treatment plan at home, χ^2^(3) = 8.06, *P* = 0.045.

Independent sample *t* tests revealed no significant group differences in change commitment or change efficacy for organizational readiness to improve the prevention and management for children undergoing surgery between children’s tertiary/quaternary hospitals (change commitment: *M* = 1.70; SD = 0.60; change efficacy: *M* = 2.07; SD = 0.75) and other health care institutions (change commitment: *M* = 1.92; SD = 0.69), *t*(82) = 1.54, *P* = 0.126 (change efficacy: *M* = 1.89; SD = 0.66), *t*(81) = −1.13, *P* = 0.264.

## Discussion

Effective perioperative pain management is essential to optimizing pediatric surgical health outcomes and preventing the transition from acute to chronic pain after surgery.^20–22^ Preventing chronic postsurgical pain is a patient, parent/caregiver, health care professional, and policymaker priority.^14,15,17–19^ Perioperative risk factors for the development of chronic postsurgical pain have been identified,^5,23^ including the risk of poorly managed presurgical and acute postsurgical pain.^6–9^ As the first phase of a project co-designing pain services to address this gap, we conducted an online survey assessing current practices for preventing and managing surgical pain at health care institutions serving children across Canada, as well as their perceived organizational readiness for change in pediatric surgical pain care. A sample of 85 health care professionals and administrators from 20 health care institutions representing varied disciplines (nursing, surgery, administration, occupational therapy, child life, physical therapy, and psychology) and institution type and size (tertiary/quaternary hospitals, and community/regional hospitals, rehabilitation hospitals, or children’s community treatment centers) responded.

Almost all participants at least somewhat agreed that pain was a priority for their institution and for surgical services. The existence of specialty pain services can be one indicator of a health care institution’s commitment to excellence in pediatric pain care.^43,44^ Most participants worked at institutions with at least one type of specialty pain service. Acute and transitional pain services can play a beneficial role in preventing the development of chronic postsurgical pain due to opportunities to intervene throughout the pre- and perioperative periods.^32,45,33,45,36,45,45,45,46^ More than 40% of participants in this study reported working at a health care institution with no acute pain service, potentially reflecting the challenge of consistently implementing a more resource-intensive specialty pain team in lower resource environments (i.e., community/regional hospitals or other children’s treatment centers).^43^ The lack of psychosocial health care professionals as members of acute or transitional pain services may identify a biomedical/pharmacological approach to acute postsurgical pain management or may reflect a lack of resource, funding, or expertise. This likely reveals a gap in care to address identified psychological risk factors in the presurgical and acute postsurgical periods for the development of chronic postsurgical pain (i.e., child and parent anxiety, pain catastrophizing, pain coping efficacy).^3,28,47^

The number of participants reporting transitional pain services at their institution was surprising given the lack of published literature about their implementation in pediatric care^25^; however, transitional pain services have seen increased adoption in adult care, with evidence supporting their value in reducing risk for chronic postsurgical pain.^31–34,48,49^ We are aware of one pediatric transitional pain service at The Hospital for Sick Children^35^; however, 70% of participants reporting transitional pain service in the current study were from other children’s health institutions, suggesting broader implementation of transitional pain services than previously known and/or services focused on preventing the transition from acute to chronic pain. It is also possible that participant responses are impacted by a lack of familiarity or consensus with the term “transitional pain service,” because other names for similar services are used (e.g., perioperative pain program,^49^ acute pain service outpatient clinic^50^) and/or ambiguity regarding the scope of transitional pain services due to potential overlap with acute or chronic specialty pain services. Furthermore, our definition of transitional pain services was not specific to surgical populations because transitional pain services may treat other medical populations at risk for the development of chronic pain and/or who would benefit from expertise in opioid stewardship (e.g., children with cancer on long-term opioids).^35^

Overall, participants reported only moderate alignment of their health care institution to 12 recommended practices^20^ for pediatric acute surgical pain care. This is notable given that close to one-quarter reported no perceived gaps. For 8 of 12 recommended practices, more than 50% of participants reported that the practice did not happen, only happened sometimes, or were unsure or did not know. In general, participants perceived best practices for postsurgical pediatric pain care to be more consistently implemented compared to presurgical pain. This may be due, in part, to specialty pain services becoming largely available during the postsurgical period. Unfortunately, results demonstrated continued inconsistent implementation of pain assessment n pain education and undertreatment of pediatric surgical pain in-hospital.^26,51^ These are critical gaps to address given the benefits of pain education and multimodal pain management to address pediatric chronic postsurgical pain psychosocial risk factors,^3,52^ as well as the health system shift to empower patients and families as partners in their own care.^53^ About 40% of participants reported that opioids were routinely prescribed for every child after surgery but, alarmingly, children and parents/caregivers received standardized information about safe use and disposal of opioids if they were prescribed less than half of the time. In many areas, perioperative pain management reported appeared comparably suboptimal to health care professional–reported practices elsewhere in the world.^54–56^

Although the majority of the most common pediatric inpatient surgeries are conducted at specialized children’s hospitals, approximately 40% are reportedly undertaken at hospitals also serving adult populations.^16^ Significant differences were noted between tertiary/quaternary children’s hospitals and other institutions (community/regional or rehabilitation hospitals and children’s community treatment centers). Surprisingly, participants from other institutions reported more consistent adherence to presurgical and most postsurgical recommendations. This is counterintuitive given that tertiary/quaternary children’s hospitals are more likely to have specialized and more resourced supports for pediatric pain care.^43^ This may be due to the larger volume and type of surgeries conducted at tertiary/quaternary hospitals (i.e., more complex, more emergency versus elective surgeries) or human resources issues making consistent implementation difficult (i.e., more staff, turnover, variability in training). Other institutions were also more likely to report routine prescription of opioids, but no significant differences were noted in the provision of information on opioid-related use and disposal. These findings cannot be accounted for by differences in the populations these health care institutions serve, because no significant differences were reported in pre- and postsurgical pain care between those institutions serving pediatric populations only and those also serving adults. More follow-up is needed to understand the implications of these differences by type of health institution, as well as adequacies in pain management given complex factors implicated in safe and effective opioid prescribing for pediatric surgical pain (i.e., dosing, delivery, monitoring, side effects, assessment).^21,22^

Overall, participants somewhat agreed that their institutions were committed and capable of change in pediatric surgical pain services. This did not differ between types of health care institutions. Change at multiple levels (health system, institution, and individual health care professional and administrator) are relevant to consider with regards to improving pediatric surgical pain care. Future research should consider what individual (e.g., health profession) and/or organizational factors contribute to perceived differences in organizational readiness for change. The Consolidated Framework for Implementation Science^57^ offers guidance for comprehensively considering factors relevant to change, including characteristics of the intervention (e.g., quality and strength of evidence, complexity, and cost of pediatric surgical pain management and service delivery models), the outer setting (e.g., patient and parent/caregivers surgical pain needs and resources, networking to other health institutions, external policies such as health standards), the inner setting (e.g., institutional culture regarding pediatric pain, leadership engagement, implementation culture), characteristics of the individuals involved (e.g., knowledge and beliefs of health care professionals about pediatric surgical pain care, self-efficacy), and the process of implementation (e.g., planning, executing, and evaluating change). Institution-wide quality improvement or knowledge translation initiatives have been successfully undertaken to improve pediatric pain^58,59^ and may offer models to guide effective change in pediatric surgical pain care. Institutions may benefit from further leveraging strengths in pediatric surgical pain care identified by participants to enact change, including perceived experience and practices of health care professionals and strong institutional commitment to pediatric pain. The lack of evidence for efficacious pain management strategies and service delivery models to prevent pediatric chronic postsurgical pain suggests a gap in both knowledge generation and knowledge mobilization.^20,25,60^

Patient partnership on the project team ensured relevance and understandability of the survey questions and provided input regarding need for exploratory analysis and interpretation of study findings. The study used a variety of recruitment methods but did not specifically recruit health care professional and/or administrator representation at each health care institution. Though this brought important diversity to the sample, individual participant knowledge of likely varies. It is possible that individuals who elected to participate include those who are more aware of key issues associated with effective pain management. The 12 recommendations for pediatric pre- and postsurgical pain care against which participants reported their institution’s current practice were drawn from existing research evidence of risk factors for chronic postsurgical pain and clinical practice guidelines^3,20^; however, we acknowledge the continued discussion regarding best practices for pediatric surgical pain, particularly related to opioids.^21,22^ Although this study went beyond tertiary/quaternary children’s hospitals, it is possible that other relevant health care institutions in Canada are not represented, particularly those that may be involved in pain management pre- or postsurgery along the continuum of care but do not conduct surgery. Future work addressing the role of services across the entire care continuum may be beneficial given that poorly coordinated care transition (i.e., between units, or transition to home) was identified by participants as an institutional gap in pediatric surgical pain care. Lastly, this study focused on health care professionals and administrators only, but patients and parents/caregivers play a critical role in pediatric surgical pain management and preventing the development of chronic postsurgical pain.^61^ Next phases of the human-centered design process^62^ of this project address this gap through interviews with patients, parents/caregivers, and health care professionals to inform multistakeholder co-design of the optimal model of pediatric pain care after surgery to prevent chronic postsurgical pain.

The study revealed the continued need to improve pediatric pain care during the pre- and postsurgical periods at Canadian health care institutions. These improvements are needed to effectively prevent the development of chronic pain in children, an expensive and debilitating health problem with lifelong consequences.^18^ This is a top priority according to patients, families, health care professionals, and policymakers alike, emphasizing the desire for urgent action.

## Supplementary Material

Supplemental Material
