# Supplementary material for: Mapping the current state of pediatric surgical pain care across Canada and assessing readiness for change
Source: Can J Pain. 2022 Jun 6;6(2):108–20. doi: 10.1080/24740527.2022.2038031 (PMC9176261; doi:10.1080/24740527.2022.2038031)
Supplement: Supplemental Material [file UCJP_A_2038031_SM7528.docx]

**Supplementary Table 1. Survey to Child Health Institutions and Health Professionals/Health Administrators about Managing Children’s Pain from Surgery**

1. Which children’s health institution do you work at or are affiliated with?
2. What is your professional role or roles? Select all that apply.
3. What clinical programs are you part of and/or do you oversee? Select all that apply.
   1. Surgical services or programs – ambulatory
   2. Surgical services or programs – major surgeries requiring inpatient stay
   3. Medical services or programs
      1. Describe: _____________
   4. Acute Pain Service (i.e., inpatient consultation)
   5. Transitional Pain Service (i.e., predominantly outpatient that serves children at-risk for developing chronic pain)
   6. Chronic/Complex Pain Service (i.e., outpatient/ambulatory multidisciplinary clinic)
   7. Intensive Pain Rehabilitation Program (i.e., outpatient and/or inpatient)
   8. Other: ______________
4. How many years have you been working in your current role? _________.
5. What is your year of birth? _________.
6. What is your sex?
   1. Female
   2. Male
7. What is your current gender identity?
   1. Female
   2. Male
   3. Trans male/Trans man
   4. Trans female/Trans woman
   5. Genderqueer/Gender non-conforming
   6. Different identity (please specify: _______________________)
8. You may belong to one or more racial or cultural groups on the following list. Select all that apply.
   1. Indigenous (e.g., Aboriginal, First Nations, Inuit, Metis)
   2. Arab
   3. West Asian (e.g., Iranian, Afghan)
   4. Black
   5. Chinese
   6. Filipino
   7. Japanese
   8. Korean
   9. Latin American
   10. South Asian (e.g., East Indian, Pakistani, Sri Lankan)
   11. Southeast Asian (e.g., Vietnamese, Cambodian, Malaysian, Laotian)
   12. White
   13. Another group: _______________
   14. Do not want to answer
9. Are you perceived or treated as a person of colour?
   1. Yes
   2. No
10. Is your children’s health institution a:
    1. Tertiary/quaternary children’s hospital
    2. Rehabilitation hospital
    3. Community/regional hospital
    4. Children's Community Treatment Centre
    5. Other: _____________
11. Does your children’s health institution:
    1. Serve pediatric populations only
    2. Serve pediatric and adult populations
12. What is the estimated number of pediatric inpatient beds at your children’s health institution:
    1. 0
    2. 1-50
    3. 51-100
    4. 100-150
    5. 151-200
    6. 200+
    7. Unsure
13. Does your children’s health institution include surgical services for children <19 years of age? Select all that apply.
    1. No
    2. Ambulatory (day) surgeries
       1. IF YES: Please indicate the approximate number of ambulatory (day) surgeries conducted at your children’s health institution per year.
          1. 1-50
          2. 51-100
          3. 101-150
          4. 151-200
          5. 200+
          6. Unsure
    3. Surgeries requiring inpatient stay
       1. IF YES: Please indicate the approximate number of major surgeries requiring inpatient stay conducted at your children’s health institution per year.
          1. 1-50
          2. 51-100
          3. 101-150
          4. 151-200
          5. 200+
          6. Unsure

**Current Pediatric Pain Care**

1. Does your children’s health institution have specialty pain consultation services or clinics? Select all that apply.
   1. Acute Pain Service (i.e., inpatient consultation)
      1. IF YES: Which health professionals are part of the Acute Pain Service? Select all that apply.
         1. Physician
         2. Nursing
         3. Psychology
         4. Physical Therapy
         5. Occupational Therapy
         6. Family Therapy
         7. Recreational Therapy
         8. Art Therapist
         9. Child Life Specialist
         10. Unsure/don’t know
         11. Other: _____________
   2. Transitional Pain Service (i.e., predominantly outpatient that serves children at-risk for developing chronic pain)
      1. IF YES: Which health professionals are part of the Transitional Pain Service? Select all that apply.
         1. Physician
         2. Nursing
         3. Psychology
         4. Physical Therapy
         5. Occupational Therapy
         6. Family Therapy
         7. Recreational Therapy
         8. Art Therapist
         9. Child Life Specialist
         10. Unsure/don’t know
         11. Other: _____________
   3. Chronic/Complex Pain Service (i.e., outpatient/ambulatory multidisciplinary clinic)
      1. IF YES: Which health professionals are part of the Chronic/Complex Pain Service? Select all that apply.
         1. Physician
         2. Nursing
         3. Psychology
         4. Physical Therapy
         5. Occupational Therapy
         6. Family Therapy
         7. Recreational Therapy
         8. Art Therapist
         9. Child Life Specialist
         10. Unsure/don’t know
         11. Other: _____________
   4. Intensive Pain Rehabilitation Program (i.e., outpatient and/or inpatient)
      1. IF YES: Which health professionals are part of the Intensive Pain Rehabilitation Program? Select all that apply.
         1. Physician
         2. Nursing
         3. Psychology
         4. Physical Therapy
         5. Occupational Therapy
         6. Family Therapy
         7. Recreational Therapy
         8. Art Therapist
         9. Child Life Specialist
         10. Unsure/don’t know
         11. Other: _____________
   5. None of the above
   6. Other: ______________
      1. IF YES: Which health professionals are part of the Other service you described? Select all that apply.
         1. Physician
         2. Nursing
         3. Psychology
         4. Physical Therapy
         5. Occupational Therapy
         6. Family Therapy
         7. Recreational Therapy
         8. Art Therapist
         9. Child Life Specialist
         10. Unsure/don’t know
         11. Other: _____________

**Current Pediatric Postoperative Pain Care**

The following questions pertain to the management of postsurgical pain. Management of chronic pain, acute nonsurgical pain, dental pain, trauma pain, and periprocedural (nonsurgical) pain are outside the scope.

1. Prior to surgery, standardized measures are completed by children and parents/caregivers to screen for risk factors for chronic postsurgical pain, including child and/or parent psychological factors (e.g., pre-existing pain, anxiety, pain coping efficacy, pain catastrophizing).
   1. Yes, with every child
   2. No
   3. Sometimes
   4. Unsure/don’t know
   5. Other comment:____________
2. Prior to surgery, children receive standardized education about pain from the healthcare team that includes information about a biopsychosocial conceptualization of pain, how to assess pain, and multimodal pain management strategies (i.e., pharmacological, psychological, and physical strategies).
   1. Yes, with every child
   2. No
   3. Sometimes
   4. Unsure/don’t know
   5. Other comment:____________
3. Prior to surgery, parents/caregivers receive standardized education about pain from the healthcare team that includes information about a biopsychosocial conceptualization of pain, how to assess pain, and multimodal pain management strategies (i.e., pharmacological, psychological, and physical strategies).
   1. Yes, with every parent
   2. No
   3. Sometimes
   4. Unsure/don’t know
   5. Other comment:____________
4. Prior to surgery, it is standard for children and parents/caregivers to be included in co-developing a postoperative pain management plan.
   1. Yes, with every child
   2. No
   3. Sometimes
   4. Unsure/don’t know
   5. Other comment:____________
5. Following surgery, standardized pain assessment tools are provided by the healthcare team to track the child’s response to postoperative pain treatments and adjust treatments accordingly.
   1. Yes, with every child
   2. No
   3. Sometimes
   4. Unsure/don’t know
   5. Other comment:____________
6. Following surgery, multimodal pain management (i.e., combining pharmacological, psychological, and physical strategies) is implemented.
   1. Yes, with every child
   2. No
   3. Sometimes
   4. Unsure/don’t know
   5. Other comment:____________
7. Following surgery, nonopioid medications (i.e., acetaminophen, nonsteroidal anti-inflammatory drugs [NSAIDs], and/or gabapentinoids) are routinely prescribed for managing the child’s pain.
   1. Yes, with every child
   2. No
   3. Sometimes
   4. Unsure/don’t know
   5. Other comment:____________
8. Following surgery, opioids are routinely prescribed for managing the child’s pain.
   1. Yes, with every child
   2. No
   3. Sometimes
   4. Unsure/don’t know
   5. Other comment:____________
9. If prescribed for postsurgical pain, children and parents/caregivers receive standardized information about safe use and disposal of opioids.
   1. Yes, with every child
   2. No
   3. Sometimes
   4. Unsure/don’t know
   5. Other comment:____________
10. Following surgery, physical interventions are routinely implemented for managing the child’s pain (e.g., physical therapy or exercise, transcutaneous electric nerve stimulation [TENS], gradual return to activity, massage).
    1. Yes, with every child
    2. No
    3. Sometimes
    4. Unsure/don’t know
    5. Other comment:____________
11. Following surgery, psychological interventions are routinely implemented for managing the child’s pain (e.g., guided imagery, distraction, music).
    1. Yes, with every child
    2. No
    3. Sometimes
    4. Unsure/don’t know
    5. Other comment:____________
12. Following surgery, children and parents/caregivers receive standardized education from the healthcare team about the pain treatment plan at home, including tapering of pain medications.
    1. Yes, with every child
    2. No
    3. Sometimes
    4. Unsure/don’t know
    5. Other comment:____________
13. What do you perceive as the strengths in pain management at your child health institution for children undergoing surgery?
14. What do you perceive as the gaps in pain management at your child health institution for children undergoing surgery?

**Readiness for Change in Pain Services for Surgery**

The following questions ask about your child health institution’s (your organization’s) readiness to make improvements in the prevention and management of pain for children and adolescents undergoing surgery.

1. Pain is a priority for our children’s health institution.
   1. Agree
   2. Somewhat Agree
   3. Neither Agree nor Disagree
   4. Somewhat Disagree
   5. Disagree
2. Pain is a priority for surgical services at our children’s health institution.
   1. Agree
   2. Somewhat Agree
   3. Neither Agree nor Disagree
   4. Somewhat Disagree
   5. Disagree
3. People who work here feel confident that the organization can get people invested in implementing this change (i.e. improving the prevention and management of pain for children and adolescents undergoing surgery).
   1. Agree
   2. Somewhat Agree
   3. Neither Agree nor Disagree
   4. Somewhat Disagree
   5. Disagree
4. People who work here are committed to implementing this change.
   1. Agree
   2. Somewhat Agree
   3. Neither Agree nor Disagree
   4. Somewhat Disagree
   5. Disagree
5. People who work here feel confident that they can keep track of progress in implementing this change.
   1. Agree
   2. Somewhat Agree
   3. Neither Agree nor Disagree
   4. Somewhat Disagree
   5. Disagree
6. People who work here will do whatever it takes to implement this change.
   1. Agree
   2. Somewhat Agree
   3. Neither Agree nor Disagree
   4. Somewhat Disagree
   5. Disagree
7. People who work here feel confident that the organization can support people as they adjust to this change.
   1. Agree
   2. Somewhat Agree
   3. Neither Agree nor Disagree
   4. Somewhat Disagree
   5. Disagree
8. People who work here want to implement this change.
   1. Agree
   2. Somewhat Agree
   3. Neither Agree nor Disagree
   4. Somewhat Disagree
   5. Disagree
9. People who work here feel confident that they can keep the momentum going in implementing this change.
   1. Agree
   2. Somewhat Agree
   3. Neither Agree nor Disagree
   4. Somewhat Disagree
   5. Disagree
10. People who work here feel confident that they can handle the challenges that might arise in implementing this change.
    1. Agree
    2. Somewhat Agree
    3. Neither Agree nor Disagree
    4. Somewhat Disagree
    5. Disagree
11. People who work here are determined to implement this change.
    1. Agree
    2. Somewhat Agree
    3. Neither Agree nor Disagree
    4. Somewhat Disagree
    5. Disagree
12. People who work here feel confident that they can coordinate tasks so that implementation goes smoothly.
    1. Agree
    2. Somewhat Agree
    3. Neither Agree nor Disagree
    4. Somewhat Disagree
    5. Disagree
13. People who work here are motivated to implement this change.
    1. Agree
    2. Somewhat Agree
    3. Neither Agree nor Disagree
    4. Somewhat Disagree
    5. Disagree
14. People who work here feel confident that they can manage the politics of implementing this change.
    1. Agree
    2. Somewhat Agree
    3. Neither Agree nor Disagree
    4. Somewhat Disagree
    5. Disagree
